# Improving Hemorrhoid Outcomes: A Narrative Review and Best Practices Guide for Pharmacists

**DOI:** 10.3390/pharmacy13040105

**Published:** 2025-07-30

**Authors:** Nardine Nakhla, Ashok Hospattankar, Kamran Siddiqui, Mary Barna Bridgeman

**Affiliations:** 1 Professional Practice Department, School of Pharmacy, University of Waterloo, 200 University Ave. W., Waterloo, ON N2L 3G1, Canada; 2 US Medical Affairs, Haleon, Warren, NJ 07059, USA; ashok.x.hospattankar@haleon.com (A.H.); or drkamran3009@gmail.com (K.S.); 3 Ernest Mario School of Pharmacy, Rutgers, The State University of New Jersey, Piscataway, NJ 08854, USA; mary.bridgeman@pharmacy.rutgers.edu

**Keywords:** hemorrhoids, astringents, rectal bleeding, anorectal discomfort, over-the-counter therapy, clinical algorithm, digital health, patient counseling

## Abstract

Hemorrhoidal disease remains a prevalent yet often overlooked condition, affecting millions worldwide and imposing a substantial healthcare burden. Despite the availability of multiple treatment options, gaps persist in patient education, early symptom recognition, and optimal treatment selection. Recent advancements are evolving the pharmacist’s role in hemorrhoid management beyond traditional over-the-counter (OTC) and prescription approaches. The 2024 American Society of Colon and Rectal Surgeons (ASCRS) guidelines introduce updates on the use of phlebotonics, a class of venoactive drugs gaining recognition for their role in symptom management, yet largely underutilized in U.S. clinical practice. In parallel, novel clinical tools are reshaping how pharmacists engage in assessment and care. The integration of digital decision-support platforms and structured evaluation algorithms now empowers them to systematically evaluate symptoms, identify red flag signs, and optimize patient triage. These tools reduce diagnostic variability and improve decision-making accuracy. Given their accessibility and trusted role in frontline healthcare, pharmacists are well-positioned to bridge these critical gaps by adopting emerging treatment recommendations, leveraging algorithm-driven assessments, and reinforcing best practices in patient education and referral. This narrative review aims to equip pharmacists with updated insights into evidence-based hemorrhoid management strategies and provide them with structured assessment algorithms to standardize symptom evaluation and treatment pathways. By integrating these innovations, pharmacists can enhance treatment outcomes, promote patient safety, and contribute to improved quality of life (QoL) for individuals suffering from hemorrhoidal disease.

## 1. Introduction

Hemorrhoidal disease remains a significant public health concern, affecting millions of individuals worldwide and imposing a substantial economic burden on healthcare systems [1]. It ranks as the third most common outpatient gastrointestinal condition in the U.S., accounting for nearly four million office and emergency department visits annually [2]. Despite its high prevalence, the true burden of hemorrhoidal disease is often underestimated due to patient reluctance to seek medical care and inconsistencies in diagnostic criteria [3]. These challenges highlight the need for improved awareness and updated management strategies to optimize patient outcomes and reduce associated healthcare costs. Historically, hemorrhoid management has relied on conservative treatments such as dietary modifications, fiber supplementation, topical astringents, and surgical interventions. While these approaches are effective for many patients, they may not be sufficient for individuals with moderate to severe symptoms. Recent advancements in pharmacologic therapies and minimally invasive procedures have expanded treatment options, necessitating an enhanced role for healthcare providers, particularly pharmacists, in patient education, symptom management, and referral processes [4].

One of the most significant developments in hemorrhoid treatment is the increased recognition of phlebotonics as a therapeutic option. These venoactive agents improve venous tone, reduce inflammation, and enhance microcirculation, making them a non-invasive alternative for patients with mild to moderate hemorrhoids [5]. Although widely used in Europe for decades, phlebotonics have only recently been acknowledged in the 2024 American Society of Colon and Rectal Surgeons (ASCRS) guidelines [6]. Despite their demonstrated efficacy in reducing symptoms such as bleeding, discomfort, and prolapse severity [7], phlebotonics remain underutilized due to limited awareness among pharmacists and inconsistencies in guideline recommendations. Addressing this gap presents an opportunity for pharmacists to play a more active role in integrating these agents into clinical practice.

In addition to pharmacologic advancements, minimally invasive procedural options such as rubber band ligation (RBL), sclerotherapy, and infrared coagulation have emerged as effective alternatives to traditional hemorrhoidectomy. These procedures offer reduced postoperative discomfort, faster recovery times, and high success rates, making them increasingly preferred for patients with grade I–III hemorrhoids [6,8]. While these treatments are typically performed in clinical settings, pharmacists must be knowledgeable about them to provide informed recommendations and guide patients in seeking appropriate interventions.

Pharmacists are increasingly utilizing structured assessment frameworks and referral algorithms to systematically evaluate symptoms, identify red flag indicators necessitating physician referral, and recommend suitable over-the-counter (OTC) or prescription treatments. These approaches strengthen patient education, improve self-care strategies, and facilitate timely medical intervention. Furthermore, technological advancements in digital health are transforming patient engagement in hemorrhoidal care. Mobile applications, telehealth platforms, and symptom-tracking tools are enhancing patient access to evidence-based guidance and remote care. By integrating these technologies, pharmacists can leverage these tools to offer more comprehensive support and optimize patient outcomes [9].

Despite these advancements, significant barriers remain, particularly the stigma associated with hemorrhoidal disease. Many patients feel embarrassed or reluctant to seek professional advice, leading to delayed treatment and worsening symptoms. Pharmacists, as highly accessible healthcare professionals, are well-positioned to address this issue by providing empathetic, non-judgmental guidance, reducing stigma, and promoting early intervention.

This narrative review aims to equip pharmacists with the latest insights into emerging treatment strategies for hemorrhoidal disease, emphasizing their expanding role in patient care. By staying informed on new pharmacologic options, structured assessment methodologies, and evolving procedural treatments, pharmacists can enhance their contributions to hemorrhoid management, ultimately improving patient outcomes and quality of life. As the field continues to evolve, integrating these advancements into pharmacy practice will be essential for delivering comprehensive and up-to-date care to individuals suffering from hemorrhoidal disease.

## 2. Methodology

This narrative literature review aimed to synthesize contemporary evidence related to the epidemiology, pathophysiology, clinical management, pharmacologic strategies, and patient-centered approaches in the treatment of hemorrhoidal disease. Articles were retrieved through targeted searches of PubMed, Embase, Medline, and Google Scholar using free-text keywords combined with Boolean operators and truncations to ensure comprehensive coverage. Keywords such as “Hemorrhoids”, “Haemorrhoid”, “Prevalence”, “Constipation”, “Pharmacist Intervention”, “Pharmacist”, “Topical Corticosteroids”, “Suppositories”, “Surgical Treatment”, “OTC Management”, “Guideline”, and “Patient Awareness” were used. Eligible studies were restricted to English-language publications, without limitations on publication type. Randomized controlled trials, observational studies, meta-analyses, clinical guidelines, product information, commentaries, and grey literature were considered. The search strategy was expanded through citation tracking to capture additional relevant literature. Full-text review and thematic analysis were performed to extract insights across domains such as disease prevalence and classification, conservative and surgical treatment modalities, patient-reported outcomes, pharmacist-driven care initiatives, and regulatory perspectives. Product labeling, FDA guidance documents, and expert narratives were integrated to enhance practical relevance and inform evidence-based decision-making by healthcare providers, particularly pharmacists engaged in the management of hemorrhoidal disease in clinical settings.

Given the broad scope and conceptual nature of the review’s objectives, a traditional systematic meta-analysis was deemed unsuitable; instead, a narrative review approach was adopted.

## 3. Epidemiology and Risk Factors

Epidemiological data indicate that determining the exact prevalence of symptomatic hemorrhoids is challenging due to several factors: (1) individuals often avoid seeking professional care, and instead depend on OTC solutions; (2) people also mistake other anorectal issues for hemorrhoids; (3) methodological limitations of various epidemiological assessment tools; (4) self-reported surveys often lack specificity, and physician-reported diagnoses or discharge data are not always confirmed [10]. Thus, epidemiologic data can vary widely.

Globally, prevalence varies widely based on self-reported surveys and physician-diagnosed cases. Recent colonoscopy studies show a prevalence of 20.8% to 38.2% [11]. In the U.S., a survey found that hemorrhoids affect approximately 4.4% of the general population, though the true incidence may be higher, as many do not seek medical care [12]. Additionally, about 1 in 20 American individuals are affected by hemorrhoids, with half of adults over the age of 50 experiencing symptoms at some point in their lives [13]. The condition is most prevalent among individuals aged 45 to 69, with similar rates observed in both men and women [13]. Additionally, studies report that white individuals were affected 1.5 times more frequently than black individuals, and individuals from higher social classes experienced hemorrhoids 1.8 times more often than those from lower classes, which may be attributed to differences in healthcare-seeking behaviors, access to medical care, and social classification systems. For instance, in England and Wales, social class was closely tied to occupation, which itself may have contributed to the observed variation in prevalence [14]. One study [14] highlights the ongoing challenges in accurately determining the true epidemiology of hemorrhoids, largely due to patients’ reluctance to seek medical attention; they often opt for self-medication out of embarrassment. Additionally, it underscores the importance of further research into the complex interplay of risk factors contributing to the development and progression of hemorrhoidal disease.

Many potential risk factors are associated with the development of hemorrhoids, including aging, obesity, prolonged straining during defecation, lower socioeconomic status, sedentary lifestyle, and depressive mood (Table 1) [11,15]. While constipation and straining are traditionally linked to hemorrhoids, recent research challenges this assumption, suggesting that diarrhea may also contribute to the risk factor of developing hemorrhoids [16]. Pregnancy is another well-documented risk factor, often leading to congestion of the anal cushions and symptomatic hemorrhoids, which typically resolve after childbirth. In addition to these factors, various dietary factors have been implicated in the development of hemorrhoids [16]. A diet low in fiber is a well-known contributor to constipation and straining, which can exacerbate hemorrhoids. Other factors, such as the consumption of spicy foods and alcohol, have also been suggested as potential contributors, although the evidence remains inconsistent [15]. More research is needed to clarify the role of these dietary elements in hemorrhoid development.

## 4. Pathophysiology, Symptoms, and Clinical Presentation

Hemorrhoid pathophysiology involves tissue degeneration leading to the downward displacement of hemorrhoidal cushions, venous dilation, and severe inflammation of the vascular wall and connective tissues. Hypotheses include mucosal ulceration, ischemia, and thrombosis. This aligns with the sliding anal canal lining theory, which suggests that degeneration of support tissues leads to venous dilation and displacement [4]. The anal canal contains two vascular arteriovenous plexuses: the internal hemorrhoidal plexus, located above the dentate line, and the external hemorrhoidal plexus. Increased pressure can cause these plexuses to become swollen and inflamed, leading to hemorrhoid formation [20]. Degeneration of collagen fibers and fibroelastic tissues within these plexuses disrupts the extracellular matrix surrounding smooth muscle, resulting in distortion and rupture of the anal subepithelial muscle [4]. This process contributes to abnormal venous dilatation and vascular thrombosis. Various enzymes and mediators that contribute to the breakdown of supporting tissues in the anal cushions have been investigated [21,22]. Matrix metalloproteinases (MMPs), particularly MMP-9, are overexpressed in hemorrhoidal tissue and contribute to the breakdown of extracellular proteins such as elastin and collagen. Neovascularization, driven by the overexpression of endoglin, is another contributing factor implicated in hemorrhoid formation, with both MMPs and endoglin being found to be overexpressed in hemorrhoidal tissue [22].

Symptoms attributed to hemorrhoids include bleeding, pain, pruritus, fecal seepage, prolapse, and mucus discharge [10]. The most common initial symptom is painless rectal bleeding during defecation. Discomfort and pain associated with prolapsing anal tissue are common reasons for patients to seek treatment, with most patients doing so within the first 12 months of experiencing symptoms [4]. Complicated hemorrhoids, like thrombosed external or strangulated internal hemorrhoids, can cause anal pain and the formation of a lump at the anal verge. Severe anal pain is more likely due to an anal fissure or anorectal abscess rather than hemorrhoids [23]. It is important to note that symptoms often attributed to hemorrhoids might have other causes. For instance, a German study found that of the 63% of patients who believed they had hemorrhoids, only 18% actually did, while the remaining patients’ symptoms were due to other conditions [24]. Therefore, identifying red flag symptoms at an early stage is crucial for accurate diagnosis. Itching is also a common symptom of hemorrhoids, often caused by poor hygiene or mucous secretion. It is thought to arise from interactions between skin nerve fibers, skin cells, inflammatory cells, and allergens penetrating the weakened skin barrier [4].

**Figure 1:**
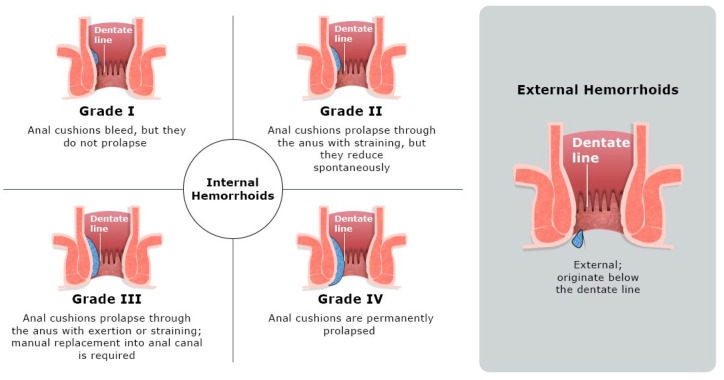
Illustrations of internal hemorrhoid grading according to the Goligher’s classification system and of an external hemorrhoid.

Although no direct correlation exists between specific symptoms and anatomic grading, the hemorrhoid classification system remains useful for selecting appropriate treatments and comparing therapeutic outcomes. Moreover, there have been attempts to create a symptom score, but a validated symptom score for hemorrhoids is not currently available [25]. Hemorrhoids are generally classified based on their location and degree of prolapse [7]. The widely used Goligher’s classification (Figure 1) distinguishes hemorrhoids based on their anatomical location into internal and external categories. Internal hemorrhoids originate from the interior hemorrhoidal venous plexus above the dentate line and are covered by mucosa. They are viscerally innervated and usually painless unless complications arise. They are further graded based on the degree of prolapse using the Goligher classification system [7]. External hemorrhoids develop below the dentate line, where they can cause pain due to the presence of nerve fibers. They are generally evaluated based on symptoms and physical examination findings, as no formal classification system, like Goligher’s, exists for them [7]. The term “mixed hemorrhoids” applies when there is bridging of internal and external plexuses [10].

Current research on hemorrhoidal disease is hindered by a lack of standardized outcome measures and validated symptom scores. This inconsistency complicates the ability to conduct meta-analyses and derive evidence-based practices in clinical settings, ultimately obstructing a comprehensive understanding of the disease. A standardized symptom assessment and diagnostic approach would significantly enhance the quality and comparability of findings across studies, leading to improved patient care and more effective treatment outcomes [26]. For example, using the Visual Analog Scale (VAS) in conditions like osteoarthritis, cancer, chronic pain, and rheumatic diseases ensures more accurate assessments and benefits clinical practice and patient management [27].

## 5. Pharmacists’ Role in Validating Patient Self-Diagnosis

Pharmacists serve as crucial frontline healthcare providers in evaluating patients with self-diagnosed hemorrhoids. Data from a study of 3812 individuals with hemorrhoidal disease revealed that of confirmed diagnoses, many were made by community pharmacists, illustrating their expanding role in frontline healthcare. As accessible professionals, pharmacists frequently engage with patients experiencing key symptoms such as pain, bleeding, and itching, often guiding initial treatment decisions and facilitating referrals when necessary [17]. This positioning makes pharmacists essential gatekeepers in distinguishing between benign hemorrhoidal disease and potentially serious colorectal conditions requiring physician referral.

The pharmacist’s evaluation process encompasses several key components: a comprehensive medical history review, symptom assessment, risk factor identification, and lifestyle evaluation. Pharmacists systematically evaluate primary symptoms (bleeding, prolapse, pain, discharge), secondary symptoms (itching, hampered anal hygiene), associated factors (self-reported toileting habits, stool frequency and consistency), risk factors (family history of cancer or polyps, comorbidities), as well as dietary and lifestyle patterns. Table 2 presents a structured assessment framework for pharmacists evaluating potential hemorrhoid cases.

**Figure 2:**
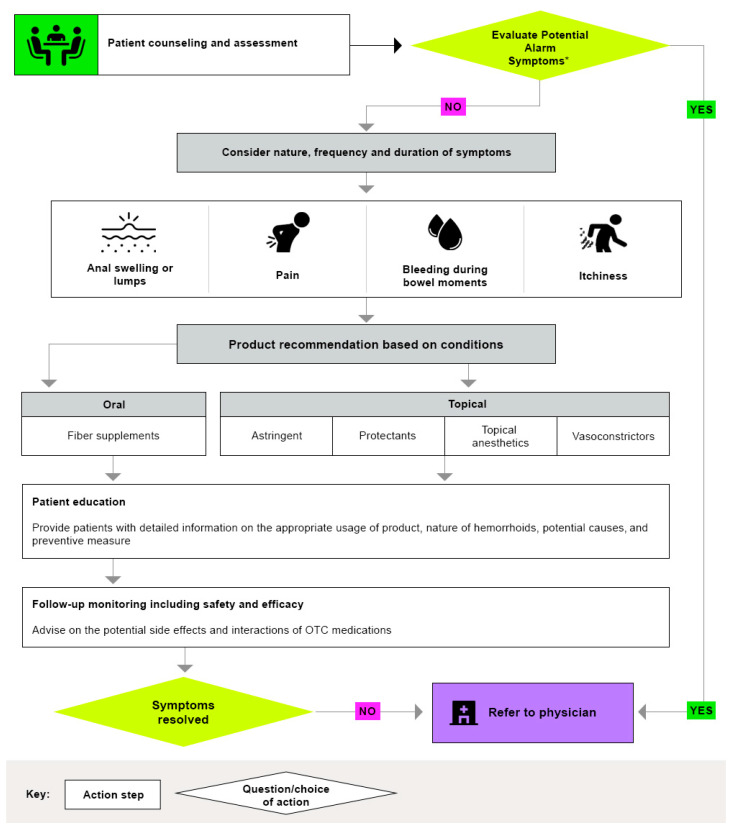
Algorithm showing the pharmacist’s role in hemorrhoid diagnosis and management. * Red flags that needed HCP attention, • If a family history of colorectal cancer, esophageal, anal cancer, • Diagnosis with a serious illness such as anal warts, anal fissures, pruritus anil IBC), perianal abscess, pinworms, polyps, proctitis and skin tags, • Non-responsive to prior hemorrhoid treatment, • Alarming symptoms including progressive unintentional weight loss, evidence of iron deficiency anemia (fatigue, breathlessness, low hemoglobin, or pale skin), change in stool frequency or consistency, gastrointestinal bleeding; Abbreviations: IBD: Inflammatory bowel disease; OTC: Over-the-Counter; HCP: Healthcare.

Figure 2 provides a step-by-step guide to the systematic approach pharmacists should adopt when evaluating hemorrhoid-related complaints:

1. Initial symptom assessment;
2. Red flag screening;
3. Treatment pathway determination;
4. Referral criteria evaluation.

Pharmacists must maintain vigilance for symptoms suggesting more sinister pathology. Key “red flag” or alarm symptoms that necessitate referral to another healthcare professional for further diagnostic evaluation are mentioned in Table 1. These symptoms or risk factors may be indicative of colorectal cancer or other conditions. Research demonstrates that hemorrhoids frequently coexist with more serious conditions. Studies have documented increased hemorrhoidal treatment prescriptions in the year preceding rectal cancer diagnosis, underscoring the need for thorough evaluation [28]. Symptoms mimicking hemorrhoidal disease, like rectal bleeding or prolapsing masses, may indicate colorectal cancer, anal cancer, anal warts, anal fissures, IBD, perianal abscess, pinworms, polyps, proctitis, and skin tags [29]. Therefore, timely medical follow-up is crucial to avoid delayed diagnosis. Patients with these symptoms, particularly those at risk for colorectal cancer, require further investigation, such as flexible sigmoidoscopy or colonoscopy, to rule out proximal pathology [30].

Certain patient groups require modified assessment and management approaches, as they are at higher risk for complications. This category includes immunocompromised patients, individuals on anticoagulation therapy, pregnant/postpartum women, and older adults (≥65 years) [30].

When conservative management fails patients, referral to primary care for more advanced interventions is warranted. This includes office-based procedures (e.g., rubber band ligation, sclerotherapy, infrared photocoagulation) that are effective for less severe cases, and surgical interventions (e.g., hemorrhoidectomy, stapled hemorrhoidopexy) or Doppler-guided hemorrhoid artery ligation for more severe cases [4,23].

## 6. Pharmacist Guide for Evidence-Based Approaches to Hemorrhoid Management

Several national and international guidelines recommend treating hemorrhoids primarily through lifestyle and dietary changes. Maintaining regular bowel movements and facilitating easy defecation can help prevent hemorrhoid prolapse and reduce bleeding, specifically in the early stages (Goligher’s degrees I and II) [31]. In addition to these changes, pharmacological treatments, such as topical ointments and phlebotonics drugs, may effectively relieve symptoms during the early stages of hemorrhoid disease. Most of these medications are available OTC; however, a few require a prescription. Prescription medications are indicated for severe hemorrhoidal symptoms, such as substantial bleeding or thrombosis. A healthcare provider customizes treatment based on the hemorrhoid type and severity, monitors the therapeutic response, and adjusts the regimen as necessary. However, in more advanced stages (III and IV), these treatments typically serve as interim measures before surgical intervention becomes necessary, acting as bridge therapy while waiting for more aggressive surgical management [32].

### 6.1. Dietary and Lifestyle Changes

Studies show that patients with constipation, straining while on the toilet, and abnormal bowel habits (e.g., prolonged sitting, frequent bowel movements) are more likely to develop symptomatic hemorrhoids. A higher prevalence of constipation has been noted among hemorrhoid patients compared to controls [12].

Increasing dietary fiber represents a primary therapeutic approach in hemorrhoid management. Evidence supports its efficacy in improving constipation and reducing hemorrhoidal bleeding, with meta-analytic data from seven clinical trials demonstrating an approximate 50% reduction in bleeding episodes. While fiber may also alleviate pruritus associated with fecal soilage, the evidence for this benefit is less robust than for bleeding reduction [33].

Current U.S. Food and Drug Administration (FDA) guidelines and the American Dietetic Association recommend specific daily fiber intake targets: for adults aged 19–50, women should consume 25–28 g per day, while men should aim for 38 g per day [34]. For adults over 50 years, recommendations are 21 g daily for women and 30 g for men. To optimize tolerability, a gradual increase in fiber consumption is advised, accompanied by adequate fluid intake of approximately 2 L of water daily [35]. Providing patients with information on the fiber content of common foods can facilitate dietary modification. When dietary sources are insufficient, commercial fiber supplements, including psyllium, polycarbophil, or wheat dextrin, may be utilized [36].

Establishing healthy bowel habits, such as avoiding straining and limiting time on the toilet, is also crucial in managing hemorrhoid symptoms. Patients are often advised to refrain from bringing distractions, such as phones or reading materials, into the bathroom [37]. Improving anal hygiene can further alleviate symptoms like itching and irritation [37]. Sitz baths, which have been shown to alleviate symptoms in pregnant women, may benefit other patients as well. Additionally, regular moderate-intensity physical activity (at least 150 min per week) has been associated with a reduction in hemorrhoid occurrence [20,23].

### 6.2. Pharmacologic Treatments—OTC Medications

Pharmacologic treatments for hemorrhoids primarily provide symptomatic relief rather than addressing the underlying cause. These treatments are broadly categorized based on the route of administration: local or topical therapies and oral or systemic treatments.

Topical products are available in various dosage forms, including creams, gels, sprays, wipes, and suppositories, allowing individuals to choose based on their preference. While these treatments aim to alleviate symptoms, they often require adjunctive therapy based on the severity of the condition [6]. Despite their widespread use, there is limited published research on the effectiveness and safety of specific treatments [38]. Many products combine multiple FDA-approved ingredients, making it difficult to assess their individual efficacy. These medications generally fall into several categories, including astringents, skin protectants, topical anesthetics, corticosteroids, analgesics, and vasoconstrictors. Oral treatments for hemorrhoids mainly include phlebotonics that are reported to improve venous tone and reduce inflammation [6,39].

#### 6.2.1. Topical Treatments

a. Astringents

Astringents (e.g., witch hazel (*Hamamelis virginiana* L.), calamine) are commonly used to alleviate itching and irritation by causing protein coagulation on the skin. Although limited clinical studies specifically address the use of astringents for hemorrhoids [40,41], a recent survey found significant improvements in QoL for patients using a sucralfate ointment containing witch hazel, measured by Haemorrhoid and Fissure Quality of Life Questionnaire (HEMO-FISS-QoL) scores. The anti-inflammatory and astringent properties of witch hazel likely contribute to these positive effects [40].

b. Skin protectants

Skin protectants (e.g., zinc oxide, glycerin, cocoa butter) are frequently used in hemorrhoid treatments to create a barrier around the afflicted area, reducing further irritation and promoting healing [42]. Cocoa butter is recognized by the FDA as a skin protectant in hemorrhoidal products under the OTC Monograph. Its inclusion in approved formulations highlights its role in soothing and shielding anorectal tissue. Products like hemorrhoidal suppositories contain up to 88.44% cocoa butter, which acts as a barrier and emollient, helping to relieve pain, itching, and burning by coating the inflamed anorectal surface [43]. They are especially useful in preventing allergens and microbes from further penetrating the compromised epidermis [44]. Skin protectants are usually formulated as creams or ointments, and provide a physical barrier that prevents allergens and microbes from penetrating the epidermal barrier [45]. Other protectants include glycerin, mineral oil, white petroleum, aluminum hydroxide gel, hard fat, kaolin, lanolin, and topical starch [46]. Although specific data on their effectiveness in hemorrhoid management are limited, these agents are considered safe and are frequently used in managing conditions like diaper rash [45].

c. Topical anesthetics

Topical anesthetics (e.g., lidocaine, pramoxine, benzocaine, benzyl alcohol, dibucaine (cinchocaine), tetracaine) are commonly used for external hemorrhoid relief [37,46]. These agents provide temporary pain relief for hemorrhoids; they can be found in a variety of OTC creams and ointments, some of which also contain ingredients that help reduce swelling and irritation. For instance, Marsicano et al. (1995) exhibited that the formulation containing lidocaine, along with calcium dobesilate and dexamethasone acetate, resulted in faster relief from hemorrhoidal symptoms, highlighting lidocaine’s pivotal role in local anesthetic action and symptom control [47]. This lidocaine-based formulation highlights its localized pain relief, though sensitivity concerns have steered some toward alternatives. Notably, pramoxine is often preferred because its unique structure lacks an ester or amide group, reducing the risk of contact dermatitis and cross-sensitivity associated with other anesthetics like lidocaine and benzocaine [48].

d. Corticosteroids

Anti-inflammatory agents like hydrocortisone bind to glucocorticoid receptors, inhibiting various inflammatory pathways and reducing mast cell activity and vasoconstriction. In the U.S., 1% hydrocortisone cream is a safe and effective OTC treatment for hemorrhoid symptoms when used as directed for up to 7 days [6]. Knoch et al. compared phytogenic preparations containing hydrocortisone with a vegetable-based ointment for hemorrhoids and found them to be highly effective [49]. Similarly, a study comparing topical hydrocortisone cream with Hai’s Perianal Support (HPS) in pregnant patients with symptomatic hemorrhoids found that both groups were effective in reducing inflammation-related symptoms such as pain, swelling, and itching [50]. Hydrocortisone is often combined with other treatments, and the FDA recommends its short-term use for relieving symptoms like pain, itching, and swelling [51]. However, prolonged use of corticosteroids can lead to adverse effects, including skin thinning, increased allergen penetration, and exacerbation of itching. Additionally, hydrocortisone may mask infection symptoms, potentially worsening conditions like tinea incognito or folliculitis [52].

e. Vasoconstrictors

Vasoconstrictors (e.g., phenylephrine) temporarily relieve symptoms. Phenylephrine is a synthetic catecholamine that stimulates alpha-1 adrenergic receptors, resulting in vasoconstriction, which helps to reduce swelling, pain, itching, and discomfort associated with hemorrhoids [46]. Phenylephrine (0.25%) is commonly used in nonprescription hemorrhoid treatments and is available in cream, ointment, wipe, suppository, and spray formulations. These products temporarily shrink hemorrhoidal tissue and reduce swelling due to irritation in hemorrhoids [53]. The U.S. FDA-approved labeling for phenylephrine-based products highlights its role in shrinking hemorrhoidal tissue and protecting irritated anorectal surfaces, which can make bowel movements less painful [54].

#### 6.2.2. Oral Treatments

a. Phlebotonics

Phlebotonics, primarily plant-based bioflavonoids, are believed to enhance venous tone, stabilize capillary permeability, facilitate lymphatic drainage, and have anti-inflammatory effects [5,39]. These drugs treat both acute and chronic hemorrhoids, though their exact mechanism is unclear [6]. A Cochrane review reported significant improvements in pruritus, bleeding, post-hemorrhoidectomy bleeding, discharge, and overall symptoms. However, their efficacy in treating external hemorrhoid thrombosis has not yet been evaluated. The European Society of ColoProctology (ESCP) guidelines recommend using phlebotonics as a primary treatment option for hemorrhoids, especially in patients with mild to moderate symptoms [8]. However, these agents are not specifically approved by regulatory agencies like the FDA for the treatment of hemorrhoids and are often used off-label due to their potential benefits. Recently, the ASCRS has acknowledged the role of phlebotonics in managing hemorrhoids, albeit with a weak recommendation due to limited long-term data. This recent recognition highlights a shift toward more formal endorsement of phlebotonics in clinical practice guidelines [6].

b. Analgesics:

The European Society of ColoProctology (ESCP) guidelines state that Nonsteroidal Anti-Inflammatory Drugs (NSAIDs) and non-opioid analgesics can be prescribed for pain management in the treatment of hemorrhoids [8]. While there is limited scientific research specifically assessing the effectiveness of NSAIDs and their derivatives in treating hemorrhoids, existing studies suggest that NSAIDs can effectively manage postoperative pain following excisional hemorrhoidectomy. A study by Rahimi et al. compared the effectiveness of EMLA cream (a topical anesthetic) and diclofenac suppository for pain relief after hemorrhoidectomy. The results indicated that both treatments offered pain relief, but EMLA cream provided better pain control in the immediate postoperative period, while diclofenac suppository was more effective for sustained pain relief [55].

### 6.3. Pharmacologic Treatments—Prescription Medications

Pharmacists play a vital role in patient care by directing individuals to healthcare providers for prescription medications when symptoms persist. These prescriptions may include potent corticosteroids, local anesthetics, venotonic agents, or analgesics, offering more effective relief than OTC options. Higher concentrations of corticosteroids or venotonic agents (phlebotonics) are available only through prescription for more serious conditions. In the context of hemorrhoid management during pregnancy, where treatment options are often limited due to concerns about fetal safety, the study [56] provides compelling evidence for the use of Proctofoam-HC, a foam-based topical medication containing 1% hydrocortisone acetate and 1% pramoxine hydrochloride. Conducted with 88 pregnant participants, the study demonstrated that this formulation significantly alleviated common hemorrhoidal symptoms such as pain, itching, and swelling, even after accounting for placebo effects (*p* < 0.001). Importantly, the treatment was also found to be safe for the fetus, with no adverse outcomes reported, making it a valuable option for symptomatic relief in late pregnancy [56,57]. Similarly, therapy containing micronized purified flavonoid fraction, a phlebotonics drug, is found to be beneficial for relieving hemorrhoidal symptoms in the majority of patients [58]. A few other prescription options for managing rectal pain associated with hemorrhoidal pain include topical nitroglycerin [59], topical nifedipine [58], and botulinum toxin injection [59], each offering effective relief through different methods.

### 6.4. Recommendations for Office-Based and Surgical Treatments

Clinicians may recommend office-based procedures for patients unresponsive to conservative treatment. These office-based procedures are effective first-line options for managing symptomatic grade I and II hemorrhoids, as well as select grade III cases. Key procedures include RBL, injection sclerotherapy, infrared coagulation, cryotherapy, radiofrequency ablation, and laser therapy. RBL shows superior efficacy and reduced follow-up procedures compared to both sclerotherapy and infrared coagulation. However, it is associated with a higher likelihood of post-procedural discomfort. A meta-analysis highlights that RBL provides significantly better outcomes than sclerotherapy, with comparable complication rates and fewer follow-up requirements. Conversely, patients undergoing sclerotherapy or infrared coagulation often require additional treatments [60].

Surgical intervention is usually required for certain patients with external hemorrhoids or those experiencing symptomatic combined internal and external hemorrhoids (grades III–IV), especially for individuals who have failed at, cannot tolerate, or are not suitable candidates for office-based procedures. Surgical intervention for hemorrhoids includes procedures like excisional hemorrhoidectomy (removal of hemorrhoids), stapled hemorrhoidectomy (repositioning hemorrhoids), and HAL-RAR (ligating hemorrhoidal arteries), each with varying recovery times and potential complications. To minimize or avoid post-hemorrhoidectomy pain, several recent surgical approaches have been adopted, which include Ligasure Hemorrhoidectomy [61], Doppler-Guided Hemorrhoidal Artery Ligation (DGHAL) [62], and stapled hemorrhoidopexy [63]. These methods aim to reduce pain and improve recovery outcomes for patients undergoing hemorrhoid surgery. DGHAL effectively treats second- or third-degree hemorrhoids but may not relieve prolapse symptoms in advanced cases. Studies show favorable short-term outcomes, but recurrence rates vary, particularly for grade IV hemorrhoids. HAL generally causes less postoperative pain than stapled hemorrhoidopexy but may have higher recurrence rates. Stapled hemorrhoidopexy is not recommended as a first-line treatment for internal hemorrhoids due to limited effectiveness and significant risks [63].

## 7. Treatment Selection and Appropriate Use: Guidance from the Pharmacist

Patient education is crucial in managing hemorrhoids, and pharmacists play a key role in this process.

### 7.1. Confirming Eligibility for Self-Treatment and Advising the Most Suitable Strategy

Pharmacists assess symptom severity to determine the appropriateness of self-treatment or referral to a physician. They play a significant role in guiding patients to select treatments based on symptoms and individual preferences [64]. Hemorrhoid treatments include medicated wipes, creams, ointments, suppositories, and sprays, often containing various combinations of active ingredients; each target specific symptoms.

Choosing the right formulation often comes down to personal preference, except in the case of suppositories, which are not suitable for external hemorrhoids. Suppositories can slip into the rectum, where they may melt, leak, or be expelled, making creams and ointments a better choice for external hemorrhoids [65].

Pharmacists must also ensure that patients are not allergic to any recommended treatment ingredients. Table 3 provides an overview of active ingredients and product forms from various classes used to treat specific hemorrhoid symptoms, and represents OTC treatment categories included as part of a pharmacist’s guidance document.

### 7.2. Ensuring Appropriate Usage

Pharmacists should ensure that patients carefully follow the product label instructions and heed any label warnings to avoid inappropriate use. Improper usage or disregarding instructions may lead to suboptimal relief or exacerbation of symptoms. Most OTC hemorrhoid treatments are labeled for external or intrarectal use only, and patients should be guided accordingly.

### 7.3. Informing About Risks

Pharmacists should educate patients on potential risks and key considerations when using hemorrhoid medications. Patients must pay attention to label warnings, especially for those with underlying conditions such as heart disease, high blood pressure, thyroid disorders, depression, diabetes, or prostate-related urination issues. If symptoms worsen, or fail to improve within 7 days, or bleeding occurs, pharmacists should advise patients to discontinue use and seek medical attention to rule out more serious underlying conditions. Additionally, they should advise patients on the maximum recommended duration for using OTC medications. For instance, prolonged use of corticosteroid-containing products should be avoided to prevent potential adverse effects.

### 7.4. Offering Guidance on Self-Monitoring and Follow-Up

Pharmacists serve as essential healthcare providers in the evaluation of symptoms self-identified as hemorrhoidal disease, guiding patients toward appropriate treatment or medical referral. Their role extends beyond immediate care to encompass several crucial aspects of ongoing patient support.

In the realm of symptom monitoring, pharmacists guide patients in systematic documentation of their symptoms, teach recognition of improvement or deterioration patterns, and establish clear parameters for seeking additional medical attention. Their expertise ensures proper medication use, addresses adherence barriers, and promotes lifestyle-based management through evidence-based education based on their diverse backgrounds and health literacy levels. The pharmacist’s role in follow-up care is equally vital, involving structured follow-ups and clear pathways for escalation of care when needed, while maintaining open communication channels for patient questions and concerns.

As key liaisons between patients and other healthcare providers, pharmacists facilitate continuity of care through systematic documentation and communication. Their position enables them to provide personalized health coaching while monitoring treatment outcomes. This comprehensive approach empowers patients to actively participate in their healthcare management, leading to optimal clinical outcomes [67].

### 7.5. Role of Pharmacists in Digital Hemorrhoid Care

In the realm of hemorrhoid care, pharmacists can also play significant role by harnessing digital platforms to enhance patient services by employing precision medication. Digital tools enable pharmacists to systematically assess symptoms, evaluate the severity of conditions, and personalized treatment plans that can be effectively managed with a combination of pharmacological interventions and dietary changes. The integration of telemedicine further expands pharmacists’ roles, allowing them to work collaboratively with other healthcare providers during remote consultations. For instance, by leveraging telehealth platforms like TMD Telehealth [68], pharmacists enhance accessibility, privacy, and the quality of care for hemorrhoid patients, contributing to better health outcomes and patient satisfaction.

Mobile health (mHealth) apps like MyTherapy and Symple allow pharmacists and patients to track symptoms, manage medications, and monitor lifestyle factors such as diet and bowel habits, which directly impact hemorrhoid severity [9]. These tools promote individualized care through progress tracking, reminders, and direct communication with healthcare providers, facilitating treatment adjustments based on patient-generated data. For example, the Symple app enables patients to log symptoms like pain or swelling while tracking their daily activities and dietary choices. Similarly, the “Hemorrhoid Tracker” app allows users to record their symptoms, dietary habits, and responses to treatments, offering critical data that can be leveraged to adjust treatment plans [9].

In addition to treatment, digital platforms also enable pharmacists to provide robust follow-up care by remotely monitoring patients’ progress and making necessary adjustments to their treatment plans. By integrating these technologies, pharmacists improve privacy, personalization, and timely care, reducing the need for in-person visits and enhancing treatment outcomes.

## 8. Limitations and Future Directions

### 8.1. Limitations of This Narrative Review

Although we performed an extensive literature search, this article is a narrative rather than a systematic review, so the selection of studies may be subject to publication and language bias. We restricted our search to English-language, peer-reviewed sources indexed in PubMed and Embase up to 1 March 2025 and did not carry out duplicate independent screening or a formal risk-of-bias appraisal. Grey literature, conference abstracts, and non-English guidelines may therefore be under-represented. Heterogeneity in study design and outcome definitions precluded a quantitative meta-analysis; consequently, recommendations are based on qualitative synthesis and should be interpreted with caution.

### 8.2. Limitations of the Available Evidence/Practice Environment

A major obstacle to advancing pharmacists’ impact in hemorrhoidal disease management is the scarcity of symptom-severity scoring tools and standardized outcome measures that have been rigorously validated for use in community practice. Without such tools, it is difficult to compare interventions, generate real-world evidence, or craft pharmacist-specific interventions. Moreover, the presence of standardized symptom-scoring tools and validated outcome measures are vital for distinguishing uncomplicated hemorrhoids from serious conditions requiring a referral [6,8]. Existing indices—e.g., the Haemorrhoid Severity Score and the Goligher grade for prolapse—were developed for surgical or specialist settings and have not been adapted to the pharmacy workflow. The resulting evidence gap is compounded by patient reluctance to disclose anorectal symptoms because of embarrassment or the misperception that hemorrhoids are trivial, leading to under-reporting and delayed care. Future work should therefore prioritize the following:

#### 8.2.1. Tool Development and Validation

Create a brief, pharmacy-compatible symptom assessment instrument that captures bleeding frequency, pain intensity, prolapse grade, and health-related quality of life, then validate it in diverse community settings.

#### 8.2.2. Digital Integration

Embed that tool in mobile apps or web portals linked to clinical decision-support algorithms. A private, app-based interface can reduce stigma while enabling longitudinal symptom tracking and personalized treatment prompts.

#### 8.2.3. Targeted Continuing Education

Offer accredited micro-learning modules and case-based workshops that (a) disseminate the 2024 ASCRS recommendations, (b) familiarize pharmacists with emerging therapies such as phlebotonics, and (c) reinforce structured assessment frameworks.

#### 8.2.4. Pragmatic Implementation Research

Conduct community–pharmacy trials—ideally randomized or stepped-wedge designs—to evaluate clinical effectiveness, cost-effectiveness, and patient-reported outcomes (quality of life, satisfaction, adherence) generated by the new tools and educational interventions.

By systematically addressing these limitations and research priorities, pharmacists can consolidate their frontline role in conservative and minimally invasive hemorrhoid care, ultimately improving patient outcomes and strengthening the evidence base for pharmacist-led practice.

## 9. Conclusions

Hemorrhoids represent a prevalent affliction among the adult population, with a wide array of OTC and prescription products available for managing acute, early-stage symptoms. Most ingredients discussed are listed in the FDA monograph of anorectal products for OTC use in hemorrhoid treatment and are generally recognized as safe and effective for temporary relief of symptoms. Lifestyle and dietary changes such as increased fiber intake and proper hydration are strongly recommended for long-term management and prevention. However, long-term management should include dietary and lifestyle changes like increased fiber intake and proper hydration.

Pharmacists play a vital role in the initial assessment and triage of patients with suspected hemorrhoids. By staying informed and integrated into the care team, they help bridge an important gap in patient management. They can confirm self-diagnosis, offer evidence-based self-care advice, and refer patients to physicians when needed, especially in cases involving red flag symptoms such as persistent bleeding, unexplained weight loss, or family history of cancer. Importantly, they can help distinguish hemorrhoids from more serious conditions like IBD or cancer. They can offer comprehensive support on a range of interventions, including medication recommendations, dietary adjustments, and lifestyle changes, which can significantly alleviate symptoms and reduce the likelihood of recurrence. Additionally, culturally sensitive education is crucial for managing such conditions, where stigma and diverse risk factors can hinder care. Tailored messaging that respects dietary habits, beliefs, and literacy levels drives better understanding and adoption. Without this inclusive approach, we risk missed opportunities and disengaged patients.

Moreover, the incorporation of advanced digital tools further complements the pivotal role of pharmacists, representing a significant breakthrough in enabling faster, more accurate, and accessible approaches to symptom evaluation and patient care. As trusted healthcare professionals within their communities, pharmacists are uniquely positioned to help patients navigate complex decisions related to self-care and the appropriate use of nonprescription treatments for hemorrhoids.

## Figures and Tables

**Table 1:** Epidemiologic characteristics of hemorrhoid prevalence by region and risk factor.

Region	Risk Factor Identified	Method of Identification	Sample Size (N)	Overall Point-Prevalence (%)	Selected Risk Factor Strata (Prevalence or Adjusted Effect)
Eight-country web survey (Brazil, Czech Republic, France, Hungary, Italy, Romania, Russia, and Spain) [17]	Sex, pregnancy, comorbidities, age, income	Self-reported	16,015	11	History of pregnancy was identified as potential risk factor associated with hemorrhoidal disease, i.e., 81% vs. 68% in the general female population Females were slightly more represented in the disease cohort (56% vs. 52%) Obesity prevalence was higher (21% vs. 19%) Mean age was greater in the disease group (46.8 vs. 44.9 years) Comorbidities were more frequent (3.1 vs. 1.3 per person) Higher income was associated with greater prevalence (38% vs. 32%) Lower income was less common among affected individuals (19% vs. 26%)
U.S. and UK national surveys/claims [14].	Age, race, sex, socioeconomic status	Surveys/admin	Multiple national datasets	4.4	Peak prevalence in 45–65 age range; rare < 20 years; declines > 65 years both sexes The overall prevalence of hemorrhoids was 1.5 times greater in white individuals than in black individuals (*p* < 0.01) Prevalence of hemorrhoids was significantly correlated with increasing social class (1.8 times more common).
U.S. and Puerto Rico [15]	Constipation, diet, pregnancy	Colonoscopy	2813	38.0 (procedure-based)	Constipation was linked to 43% higher prevalence of hemorrhoids (OR = 1.43; 95% CI: 1.11–1.86) High grain fiber intake significantly lowered the risk (OR = 0.78; 95% CI: 0.62–0.98)
South Korea [18]	Age, sex, obesity, depression	Survey	17,228	14.4	Obesity (OR = 1.13; 95% CI: 1.01–1.26) and abdominal obesity (OR = 1.16; 95% CI: 1.04–1.30) were linked to increased hemorrhoid risk Depression, both self-reported (OR = 1.83; 95% CI: 1.62–2.08) and physician-diagnosed (OR = 1.71; 95% CI: 1.35–2.17), showed a strong association with higher risk Lack of regular walking raised risk modestly (OR = 1.11; 95% CI: 1.00–1.23) Pregnancy was associated with increased risk (OR = 1.62; 95% CI: 1.17–2.25)
Northwest Ethiopia [12]	Constipation, family history, comorbidities	Outpatient	403	13.1	Constipation (AOR = 4.32, 95% CI; 2.20, 8.48) and BMI ≥ 25kg/m^2^ (AOR = 2.6, 95% CI; 1.08, 6.23) had statistically significant associations with hemorrhoids
Makkah, Saudi Arabia [19]	Sex, constipation, family history	Survey	400	16	Family history of hemorrhoidal disease was linked to a higher prevalence (21.5% vs. 10.8%) (*p* = 0.004) Pregnancy significantly increased risk (39.1% vs. 7.6% in nulligravida) Low-fiber diet was associated with more cases (24.8% vs. 13.7% with balanced diet; 3.6% with low-carb diet) Chronic conditions showed higher hemorrhoid occurrence than those with no health issues

Abbreviations: OR: odds ratio; AOR: adjusted odds ratio; CI: Confidence Interval; BMI: body mass index.

**Table 2:** Structured assessment framework for hemorrhoid diagnosis and referral determination.

Assessment Domain	Key Questions	Evaluation Criteria	Quantitative Measures
Demographics	Age and sex of patient	Age > 65 warrants additional scrutiny and possible referral	
Primary Symptoms	Nature of bleeding (if present) Character of pain/discomfort Presence of lumps/swelling Associated pruritis	Bright red blood on toilet tissue Pain pattern during/after defecation Palpable perianal masses Persistent anal irritation	Frequency of bleeding episodes per week Pain intensity score (0–10) Size of masses (in cm) Duration of irritation (hours/day)
Bowel Habits	Defecation frequency Stool consistency Straining patterns	Number of bowel movements per week Frequency of excessive straining	Normal range: 3x/week to 3x/day Bristol Stool Scale (BSS) assessment—ranges from type 1 to type 7 to assess stool consistency and digestive health Types 1 and 2: Constipation Types 3 and 4: Ideal, healthy Types 5 to 7: Diarrhea Frequency of straining episodes per bowel movement
Medical History	Previous hemorrhoid diagnosis Family history of colorectal conditions Current medications Comorbidities	Special attention to the following: Cancer history in first-degree relatives; Anticoagulation therapy; Immunosuppression.	Previous diagnoses (yes/no) Current medications list Comorbid conditions list
Lifestyle Factors	Dietary fiber intake Fluid consumption Physical activity level Occupational factors	Types of fiber consumed (fruits, vegetables, whole grains) and dietary patterns Regularity and consistency in fluid intake Consistency in fiber intake over meals Desk job/physical job; active lifestyle/sedentary lifestyle	Quantitative assessment of te following: Daily fiber (g); Fluid intake (L); Activity (hours/week).
Red Flag Symptoms	Unexplained weight loss Anemia (manifesting as fatigue, breathlessness, low hemoglobin, or pale skin) Personal or family history of colorectal cancer or polyps (especially if a first-degree relative was diagnosed before age 50 or if multiple first-degree relatives are affected) Personal or family history of inflammatory bowel disease (IBD) Stool alterations (dark red or tarry stool, changes in stool frequency, shape, or consistency)	Any findings warrant immediate referral	Amount of weight loss (kg) Hemoglobin levels (g/dL) Changes in stool pattern (as described above)

**Table 3:** OTC active ingredients and product forms for hemorrhoid symptom management: a pharmacist reference table.

Class.	Mechanism of Action	Key Ingredient(s)	Combination Ingredients	Primary Symptom ^a^	Additional Benefits ^a^	Product Formulation	Dosage/Usage ^b^
Astringents	Protein coagulant effect [64]	Witch hazel	NA	Itching, discomfort, irritation, burning	NA	Wipes	Use up to 6 times daily or after each bowel movement and before applying topical hemorrhoidal treatments, and then discard.
			NA		NA	Medicated Cooling Pads	Apply externally to the affected area up to 6 times daily or after each bowel movement.
			NA		NA	Spray	
			+ phenylephrine		Swelling, shrinking of hemorrhoidal tissue	Gel	Apply externally to the affected area up to 4 times daily, especially at night, in the morning, or after each bowel movement.
Protectants	Create a barrier around the afflicted area [66]	Zinc oxide	+ precipitated sulfur + resorcinol + salicylic acid	Itching, discomfort, irritation, burning	NA	Ointment	Twice a day or as needed.
			+ cocoa butter		Relief of pain associated with bowel movement and inflamed, anorectal surface inflammation	Suppositories	Insert 1 suppository bulb shape first into the rectum up to 6 times daily or after each bowel movement.
		Glycerin	+ lidocaine + phenylephrine		Rapidly numb and relieve pain, soreness, swelling, anorectal surface inflammation, pain associated with bowel movement	Wipes	Apply externally to the affected area up to 4 times daily, and then discard.
			+ white petrolatum + pramoxine + phenylephrine		Relief of anorectal discomfort, shrinking of hemorrhoidal tissue, protect anorectal surface inflammation, relief of pain associated with bowel movement	Cream	Apply externally to the affected area up to 4 times daily, especially at night, in the morning, or after each bowel movement.
			+ white petrolatum + lidocaine + phenylephrine		Relief of anorectal discomfort, shrinking of hemorrhoidal tissue, protect anorectal surface inflammation, relief of pain associated with bowel movement	Cream	
		Mineral oil	+ petrolatum + phenylephrine		Relief of anorectal discomfort, shrinking of hemorrhoidal tissue, protect anorectal surface inflammation, relief of pain associated with bowel movement	Ointment	
		Cocoa butter	+ phenylephrine		Relief of anorectal discomfort, shrinking of hemorrhoidal tissue, protect anorectal surface inflammation, relief of pain associated with bowel movement	Suppositories	Adults: Insert one suppository into the rectum up to 4 times daily, especially at night, in the morning, or after each bowel movement.
Anesthetics	Pudendal and anal blocks [66]	Pramoxine hydrochloride	+ zinc oxide	Pain, burning, itching	Relieves soreness and irritation, soothes and lubricates	Cream	Adults and children 12 years and older: Apply externally to the affected area up to 5 times daily.
		Lidocaine ^c^	NA		NA	Cream	For adults and children 12 years and older, can be used up to 6 times daily.
			+ glycerin		Relief of anorectal discomfort and irritation, temporarily protect inflamed perianal skin	Wipes	Adults and children 12 years and older: Apply up to 6 times daily or after each bowel movement, and then discard.
			+ mineral oil + white petrolatum + phenylephrine		Reduces swelling associated with irritated hemorrhoidal tissue, relief of anorectal discomforts, protects irritated areas and inflamed perianal skin	Cream	For adults and children 12 years and older, apply externally to the affected area up to 4 times daily. Do not exceed a combined total of 4 applications per day.
			+ phenylephrine		Reduces swelling of inflamed tissue, relief of irritation	Spray	Apply externally to the affected area up to 4 times daily.
Corticosteroids	Helps reduce inflammation [66]	Hydrocortisone 1% ^c^	NA	Itching, irritation, rashes	NA	Cream	Adults and children 12 years and older: Apply externally to the affected area not more than 3 to 4 times daily.
Vasoconstrictors	Vasopressor effect on the arterial site of circulation [30]	Phenylephrine	NA	Itching, discomfort, swelling	Shrinks hemorrhoidal tissue	Suppositories	Adults: Insert one suppository into the rectum up to 4 times daily, especially at night, in the morning, or after each bowel movement.

^a^ Temporarily relieves; ^b^ see package labels for details; ^c^ higher concentrations available by prescription only; + represents the additional ingredient/ingredients along with active ingredient. NA: not applicable.

## Data Availability

No new data were created or analyzed in this study.
